# P2Y12 receptor-mediated microglia activation involved in delayed encephalopathy after acute carbon monoxide poisoning

**DOI:** 10.18632/aging.202607

**Published:** 2021-02-20

**Authors:** Wenping Xiang, Zhigang Yang, Hui Xue, Jingbo Wang, Fanyan Niu, Jie Wang, Chao Chen, Yaming Wang, Jiangxia Pang, Baojun Wang

**Affiliations:** 1 Department of Neurology, Baotou Central Hospital, Baotou 014040, Inner Mongolia, China; 2 Department of Urology, Baotou Central Hospital, Baotou 014040, Inner Mongolia, China

**Keywords:** carbon monoxide poisoning, delayed encephalopathy, microglia, P2Y12 receptor

## Abstract

To investigate the role of P2Y12 receptor-mediated microglia activation in delayed encephalopathy after acute carbon monoxide poisoning (DEACMP), we used static inhalation carbon monoxide to build DEACMP rat model. DEACMP rats were randomly assigned into DEACMP group and intervention group. A control goup was also set. The rats in intervention group received intraperitoneal injection of 100uM suramin (a P2Y12 receptor antagonist). In control group, the escape latency, level of microglia activation and ATP content were similar between different time points. In both DEACMP group and intervention group, the escape latency, level of microglia activation and ATP content were significanlty increased at 21th and 28th day. The hippocampal cells in DEACMP group and intervention group were severely and moderately, respectively, damaged at 21th and 28th day. Meanwhile, compared to control group, both DEACMP group and intervention group had significanlty longer escape latency, higher level of microglia activation and ATP content at 21th and 28th day. Compared to DEACMP group, the intervention group had significantly shorter escape latency and lower level of microglia activation at 21th and 28th day. These results suggested that the microglia activation regulated by ATP through P2Y12 receptor pathway might be closely related to the development of DEACMP.

## INTRODUCTION

Delayed encephalopathy after acute carbon monoxide (CO) poisoning (DEACMP) is one of the most common and serious complications of CO poisoning. The incidence rate of this disease ranges from 13% to 50%, and its mortality rate could be up to 31% [1, 2]. DEACMP could result in many severe clinical symptoms, such as dementia, visceral autonomic nervous system dysfunction, Parkinson's syndrome, memory impairment and cognitive dysfunction. It brings huge economic burden to individuals, families, and society. Our previous studies found that both N-Butylphthalide and dexamethasone could significantly improve the efficacy of hyperbaric oxygenation therapy in treating DEACMP [3, 4]. But the optimal treatment methods of DEACMP are not yet identified, and the pathogenesis of DEACMP is still unclear [5].

Many factors are associated with the occurrence of DEACMP, such as the decreased hypoxia tolerance of nervous tissue in brain [6] and the prolonged duration of coma after acute CO poisoning [7]. Previous study found that DEACMP might be caused by the oxygen deficit [8]. The excitatory amino acid was also found to have a vital role in the occurrence of DEACMP [9]. Meanwhile, some researchers thought that the inflammatory reaction in blood vessel and immune damage had a close relationship with the development of DEACMP [10, 11]. Our previous study found that inflammation might play an important role in delayed encephalopathy of rats induced by acute CO intoxication [12]. From the early pathological damage of DEACMP, the immunological damage mechanism might be more likely to explain the characteristics of this disease.

Microglia, as important immune effectors of the central nervous system (CNS), can play an important role as resident immunocompetent and phagocytic cells in the event of injury or disease. Currently, the correlation between microglia activation and cognitive impairment has received wide attention. Previous studies have confirmed that microglia were the key factor in the cognitive decline of Alzheimer's disease (AD) [13, 14]. Machado et al., reported that the microglia activation-mediated neuroinflammation could play an important role in the early onset of Parkinson's disease (PD) [15]. Meanwhile, some studies reported that the morphology of microglia changed from a resting state to an activated state after CO poisoning [16, 17]. However, the potential mechanisms of CO mediating the activation of microglia are unclear.

Adenosine triphosphate (ATP) and its hydrolysate (adenosine diphosphate, ADP) are important energy carriers and also important neurotransmitters. P2 purine receptor is a physiological receptor of purine substances, such as ATP. Previous studies found that P2Y12 receptors were widely expressed in the CNS, and the positive cells were mainly microglia [18, 19]. Davalos et al., reported that the noxious stimuli could lead to the release of ATP; then both ATP and ADP could activate microglia [20]. Meanwhile, Haynes et al., found that under the stimulation of ATP released from the injured tissue, the P2Y12 receptor was activated to activate microglia, and in the P2Y12 receptor gene knockout model, no significant changes were found in the morphology of microglia; these findings suggested that the activation of P2Y12 receptors had an important role in the process of microglia activation [21]. Therefore, we conducted this study to further explore the role and function of microglia activation mediated by P2Y12 receptor in the development of DEACMP.

## RESULTS

### Manifestations of acute CO poisoning

After five to fifteen minutes of CO poisoning, the rats showed excitement and mild restlessness, followed by shortness of breath, increased breathing amplitude, cherry red color in the mucous membranes of mouth and nose, and cherry red color in the skin of ears, paws and tail. Some rats showed mania, tetraplegia, and convulsions, and some rates were unconscious or even died. The autopsy of the dead rats revealed that there were severe brain tissue edema and severe internal organ hyperemia with scattered bleeding points. After leaving the cabin, the consciousness of rats gradually recovered after inhaling air for 10 minutes.

### Cognitive function evaluation

**Figure 1:**
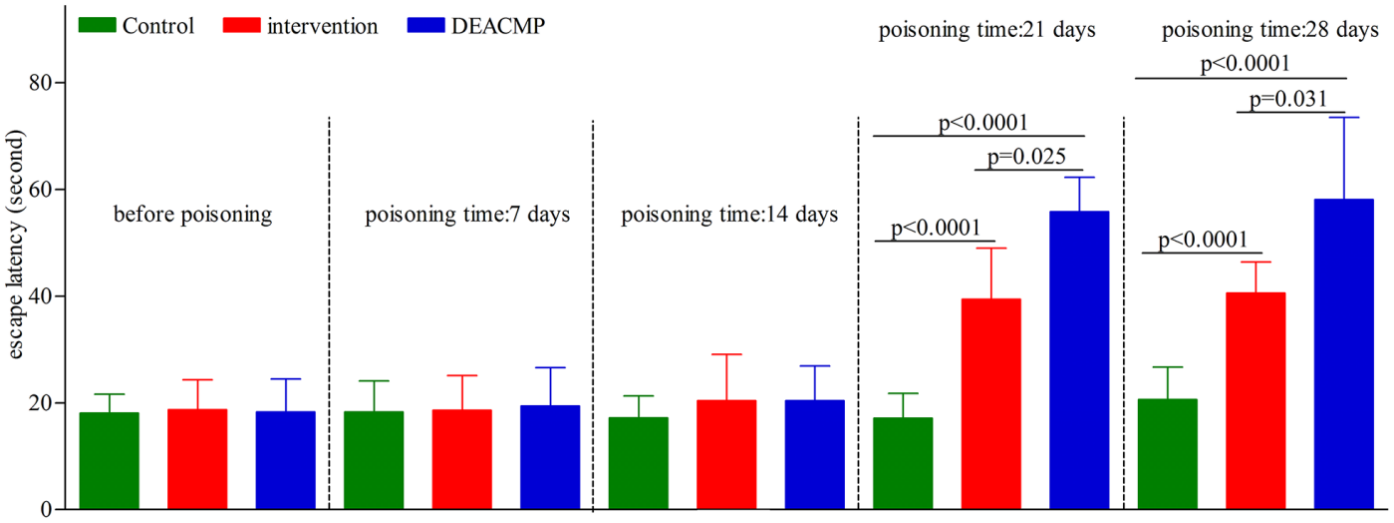
**Escape latencies of Morris water maze test in different groups.**

As shown in Figure 1, in control group, the escape latencies in different subgroups were similar to their escape latencies before poisoning. Compared to their escape latencies before poisoning, the escape latencies in DEACMP-7th day group, DEACMP-14th day group, intervention-7th day group, and intervention-14th day group were not significantly changed, but the escape latencies in DEACMP-21th day group, DEACMP-28th day group, intervention-21th day group, and intervention-28th day group were significantly increased. The escape latencies in DEACMP-21th day group and DEACMP-28th day group were significanly longer than the escape latencies in intervention-21th day group and intervention-28th day group, respectively. The escape latencies in intervention-21th day group and intervention-28th day group were significanly longer than the escape latencies in control-21th day group and control-28th day group, respectively. These results suggested that there was cognitive impairment in both DEACMP group and intervention group, but the cognitive impairment was lighter in intervention group than in DEACMP group.

### Damage of hippocampal cells

**Figure 2:**
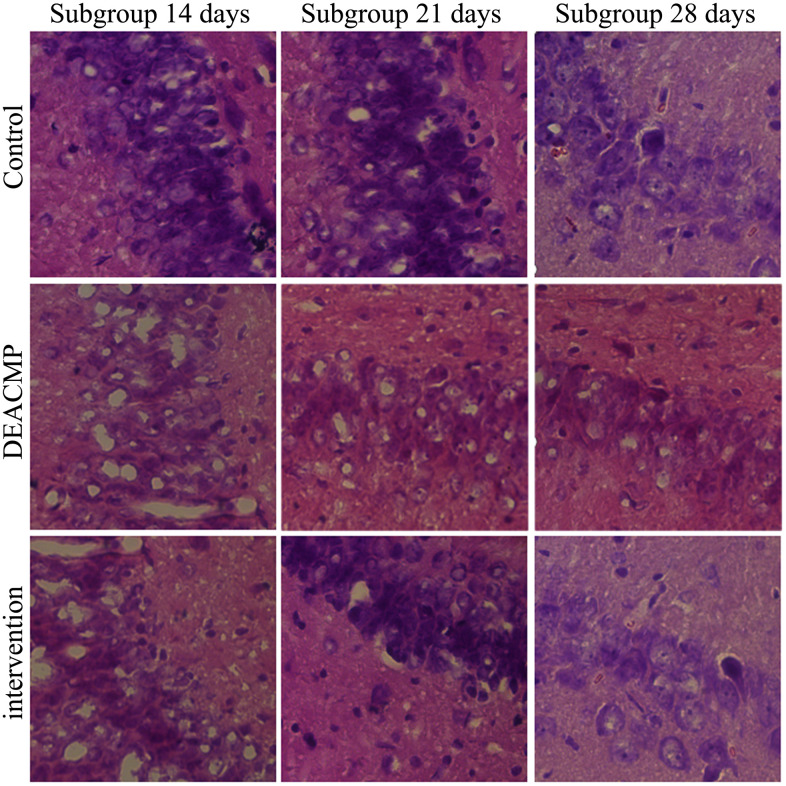
**Damage of hippocampal cells in different groups.**

The results of HE staining showed that the hippocampal neurons of rats in control group were full and with obvious perinuclear pale circles, and the cells were neatly scattered. In DEACMP group, the hippocampus was extensively damaged; the number of cells was reduced; the cell layer was thinned; the cell structure was distorted and chaotic, and the cells were obviously shrunken; but we did not observe any obvious necrotic foci. Meanwhile, the damage of hippocampal cells was observed in intervention group, but the pathological changes of the hippocampus in intervention group were significantly improved compared to DEACMP group. These phenomena could be seen in Figure 2.

### ATP content assessment

**Figure 3:**
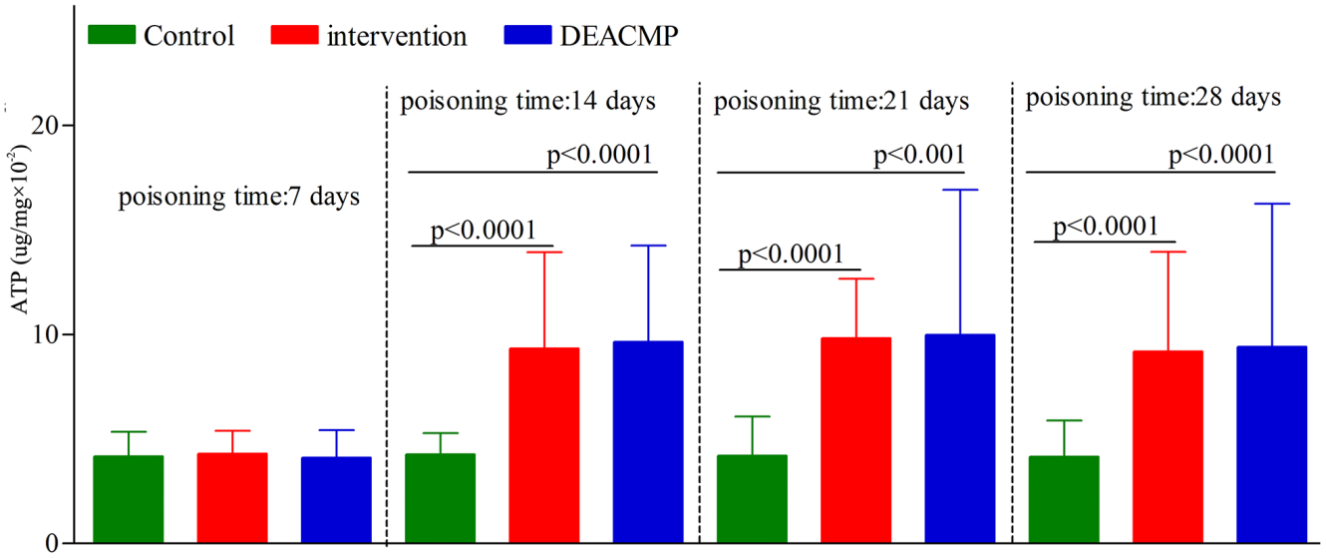
**ATP levels in different groups.**

In control group, the ATP content in different subgroups were similar (Figure 3). In DEACMP group, compared to DEACMP-7th day group, the other three subgroups had significanlty higher ATP contents (Figure 2). In intervention group, compared to intervention-7th day group, the other three subgroups had significanlty higher ATP contents (Figure 2). The ATP contents in DEACMP-14th day group, DEACMP-21th day group and DEACMP-28th day group were significanly higher than the ATP contents in control-14th day group, control-21th day group and control-28th day group, respectively (Figure 2). The ATP contents in DEACMP-14th day group, DEACMP-21th day group and DEACMP-28th day group were similar to the ATP contents in intervention-14th day group, intervention-21th day group and intervention-28th day group, respectively (Figure 3).

### Microglia activation level in the hippocampus

**Figure 4:**
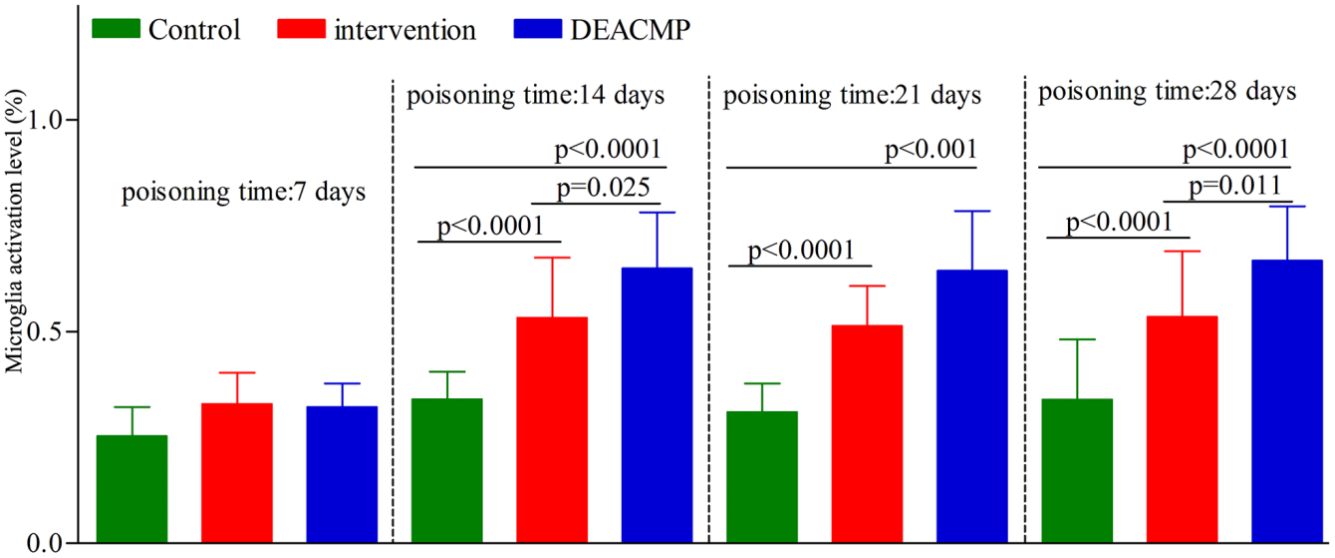
**Microglia activation level in the hippocampus in different groups.**

**Figure 5:**
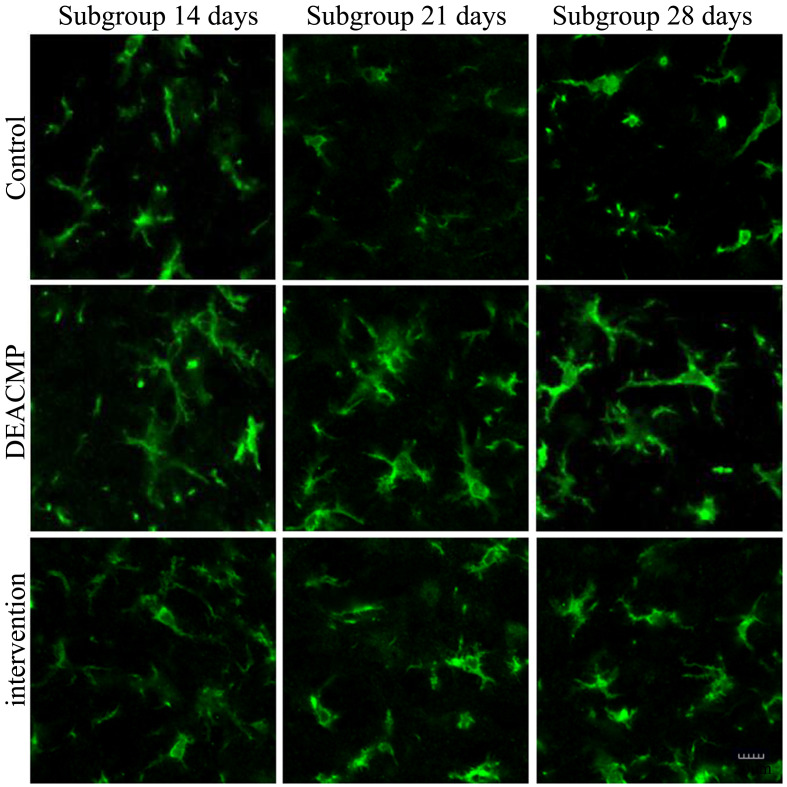
**Number of activated microglia in the hippocampus in different groups.**

As shown in Figure 4, in control group, the levels of microglia activation in different subgroups were similar. In DEACMP group, compared to DEACMP-7th day group, the other three subgroups had significanlty higher levels of microglia activation. In intervention group, compared to intervention-7th day group, the other three subgroups had significanlty higher levels of microglia activation. The levels of microglia activation were significantly higher in DEACMP-14th day group, DEACMP-21th day group and DEACMP-28th day group than in intervention-14th day group, intervention-21th day group and intervention-28th day group, respectively. The levels of microglia activation were significanly higher in intervention-14th day group, intervention-21th day group and intervention-28th day group than in control-14th day group, control-21th day group and control-28th day group, respectively. The results of immunofluorescence also showed the similar phenomenon (Figure 5).

## DISCUSSION

The key to exploring the pathogenesis of DEACMP is the successful establishment of animal model. At present, the main methods for building DEACMP rat model are CO intraperitoneal injection and CO inhalation. The main advantages of CO intraperitoneal injection are simple operation, strong controllability, and less dosage of CO [22]. But the biggest disadvantage of this method is that it is different from the clinical poisoning way. In addition, the CO absorption of this method is not constant; the rats have the high risk of death due to the sudden ingestion of a large dosage of CO. Therefore, Hara et al. recommended researchers to build DEACMP rat model using CO inhalation [23]. This method is consistent with the clinical poisoning way and has a good repeatability. In this study, we used the CO inhalation to build DEACMP rat model. Our results showed that on the days 21 and 28 after CO poisoning, compared to control group, DEACMP group showed serious cognitive impairment and severely damaged hippocampal cells, and had significantly increased level of microglia activation and ATP content in the hippocampus. Compared to DEACMP group, the intervention group showed significant improvement on cognitive function, hippocampal cellular structure and the level of microglia activation. These findings demonsrated that the microglia activation regulated by ATP through P2Y12 receptor pathway might be closely related to the development of DEACMP.

As a special type of cell in the CNS, the main functions of microglia are to provide immune support and to protect the brain tissues. It is viewed as one of the most important supporting structures of CNS. Typically, microglia activation has a pivotal role in the pathogenesis of neurodegenerative diseases. Recently, the correlation between the activation of microglia and cognitive impairment has become a research hotspot. Previous studies reported that microglia were a key factor in cognitive decline in AD [13, 14, 24]. Others found that microglia had a close relationship with the progression of PD, which was a movement disorder of CNS that could impair the cognitive functions of patients [25, 26].

Our previous study confirmed that the released inflammatory factors after CO poisoning caused the loss of myelin basic protein in the brain, and finally resulted in the onset of DEACMP [12]. In this study, the microglia in the hippocampus of rats were successfully isolated, and the expression levels of two microglia markers (CD45 and CD11b) were detected using flow cytometry; the levels of microglia activation in different groups were also analyzed. The results showed that the levels of microglia activation in the hippocampus of DEACMP rats were significantly increased on the day 14 after CO poisoning, and this phenomenon could last at least on the day 28 after CO poisoning. It demonstrated that the activated microglia were closely involved in the occurrence and development of DEACMP.

Previous study found that the peripheral sensory nerve endings could release ATP after artificial stimulation, and the authors firstly proposed that ATP might also participate in the transmission of information in the nervous system [27]. Meanwhile, Amadio et al. found that ATP could have toxic effects on cerebellar tissues or cells, and P2 receptors might be involved in the signal transduction pathway of ATP toxicity; then they deduced a hypothesis that ATP, as an important mediator, mediated the neuropathology of brain injury [28]. Normally speaking, the concentration of extracellular ATP is a relatively balanced system. But in neurodegenerative diseases, especially acute and chronic injury diseases, a large amount of ATP accumulation is often found. Here, we found the significantly increased ATP contents in both DEACMP group and intervetion group. Thus, these findings indicated that ATP and its hydrolysate (ADP) might play a role in the pathogenesis of neurodegenerative diseases.

In the present study, we found that the ATP content in the hippocampus tissue of DEACMP rats was significantly increased on the day 14 after CO poisoning, and this phenomenon could last at least on the day 28 after CO poisoning. The ATP contents were similar between DEACMP group and intervention group. But in intervention group, rats showed significantly improved cognitive function, improved pathologic change of hippocampal cells and lower level of microglia activation. These results suggested that a large amount of ATP had accumulated in the hippocampus before the occurrence of delayed encephalopathy, and the significantly increased accumulation of ATP might mediate the activation of microglia through the P2Y12 receptor pathway, thereby participating in the onset of DEACMP.

Our study mainly focused on the experimental researches on animals, and did not include cellular level researches; thus, the relevant evidence of *in vitro* experiments is temporarily lacking. The mechanisms of P2Y12 receptor mediating microglia activation and the role of activated microglia in the occurrence and development of DEACMP are needed to be further explored *in vitro*. In addition, the efficacy of P2Y12 receptor antagonist suramin could be affected by the dosage and treatment time, and further studies are needed to confirm its optimal treatment plan. However, our study firstly suggested that the microglia activation regulated by ATP through P2Y12 receptor pathway might be closely related to the development of DEACMP.

## MATERIALS AND METHODS

### Animal model

Male adult Wistar rats (6-8 weeks, 180-230g, Specific Pathogen Free) were purchased from the Animal Center of Inner Mongolia University. In total, 420 rats were used in this study, and 242 rats survived at last. The rats were housed under standard conditions: room temperature (18-23° C), 12 hours light/dark cycle, free access to water and food. This study was reviewed and approved by the Ethics Committee of Inner Mongolia University (Approved No.2016-0001). The procedures were performed according to the National Institutes of Health Guidelines for Animal Research (Guide for the Care and Use of Laboratory Animals, NIH Publication No.8023, revised 1996).

Morris water maze (MWM) task training was conducted after one week of adaptive feeding. First, the rats were placed on the escape platform for 10 seconds. Second, the rats were randomly placed into water maze from four sites to learn how to find the escape platform (four times per day). If the rat failed to find the escape platform within 2 minutes, it would be placed on the escape platform for 10 seconds. After five days, the rats that could find the escape platform within one minute were chosen for the following experiment.

The qualified rats were randomly assigned into DEACMP group, intervention group and control group. The rats in both DEACMP group and intervention group were used to build poisoning model using static inhalation CO [29, 30]. According to the results of our previous study, the opitmal concentration of CO uisng to establish poisoning model was 3000 ppm (40 minutes per day, 21 days) [31]. The rats in control group received the same amount of air. The rats in intervention group received intraperitoneal injection of 100uM suramin (a P2Y12 receptor antagonist). Both DEACMP group and control group received equal volume of normal saline.

**Figure 6:**
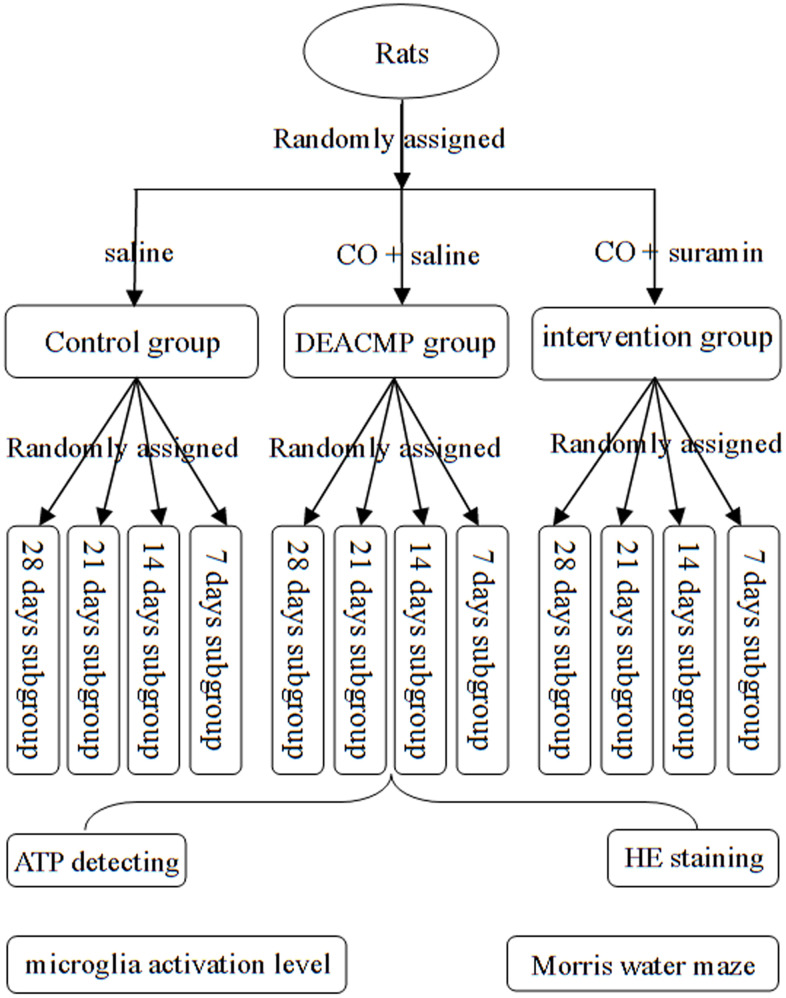
**Flow diagram of this study.**

In order to observe how the indexes (cognitive function, hippocampal cells, level of microglia activation and adenosine triphosphate (ATP) content) changed over time, the rats in DEACMP group, intervention group and control group were further randomly divided into four subgroups acorrding the poisoning time: 7th day, 14th day, 21th day, and 28th day. Each subgroup had eight rats. The animal model was successfully established if the rats met the following criteria: i) the cognitive impairment was confirmed by Morris water maze test; and ii) cell damage in the hippocampus was observed [32]. The flow diagram of this study was described in Figure 6.

### Indexes assessment

The MWM test was widely used to study learning and spatial memory. This test was carried out in each subgroup. The rats were randomly placed into water maze from four sites to find the escape platform. If the rat failed to find the escape platform within 2 minutes, the escape latency was recorded as 120 seconds. The water maze software HVS Water 2020 was used to analyze the performance of the rats. The escape latencies of rats in each subgroup were used to assess the cognitive function. Hematein eosin (HE) staining was used to evalute the damage of hippocampal cells. The HE staining steps were as following: 1) put the slices into distilled water for 5 minutes; 2) put the slices into hematoxylin for 5-10 minutes, and then rinse with tap water for 5-10 seconds to get off the floating color; 3) color separation in hydrochloric acid and alcohol for a few seconds until the observation was satisfactory under the microscope, and then rinse with tap water for 1-2 minutes; 4) return to blue in ammonia water for 5-10 seconds, and then rinse with tap water for 10-30 seconds; 5) put the slices into eosin for 1-2 minutes, and then rinse with tap water for 1-2 seconds to get off the floating color; 6) successively put the slices into 70%, 80%, 90%, 95% alcohol for 5 minutes each; 7) successively put the slices into 100% alcohol I and II for 10 minutes each; 8) transparent in xylene I and II for 15 minutes each, seal with neutral gum and observe hippocampal cells under an optical microscope. Hippocampus was a part of the brain and associated primarily with cognition and memory. Thus, it was analyzed in this study. A brain matrix was used to cut hippocampus.

The flow cytometry was used to assess the level of microglia activation in the hippocampus. Brifly, the steps were as following: 1) hippocampus tissue was cut into pieces under cold GKN (containing 0.02% bovine serum albumin), and then transferred into 50ml centrifuge tube; 2) after washing with GKN-BSA, centrifugation at 1000r/minute for 10 minutes was conducted; 3) the precipitate was digested with digestion buffer (containing 15u collagenase and 500u DNase) at 37° C for one hour; 4) after washing with GKN-BSA, centrifugation at 1000r/minute for 10 minutes was conducted; 5) the precipitate was resuspended in 4 ml 37% Percoll, and then 70% and 30% Percoll were slowly attached to the lower and upper layers, respectively; 6) after conducting centrifugation at 1000r/minute for 30 minutes and removing the supernatant, the tissue precipitate at the junction of 37% and 70% Percoll was obtained; 7) resuspend again with 0.01M PBS solution, centrifugation at 1000r/minute for 7 minutes; then collect the cell pellet and resuspend the cell pellet in 0.01M PBS solution; 8) a cell counting plate was used to count the cell number; 9) the cells were incubated with 20μg/ml CD16 Fc-Block antibody at 4° C for 20 minutes; 10) the cells were further incubated with mouse CD11b-PE and CD45-FITC fluorescent-labeled flow cytometry antibodies for 20 minutes in a dark room. The CD11b+/CD45+ (microglia) were isolated uisng American BD FACS Canto II flow cytometer.

High performance liquid chromatography was used to analyze the changes of ATP content. Briefly, a chromatographic ODS C18 column (4.6mm×250mm, 5um) was used. Mobile phase: 99% 0.18 mmol/L phosphate buffer; 1% pure methanol solution; detection wavelength: ultraviolet wavelength 254nm; injection volume: 10uL; column temperature: room temperature. The standard products were accurately weighed, and 0, 40, 80, 120, 160, 200ug/mL ATP standard solution were prepared. The 10 uL ATP standard solutions were injected under the above chromatographic conditions. The standard curve equation was obtained after performing regression processing on the peak area with the mass concentration of each group. We added 0.6M cold perchloric acid into the hippocampus tissue to make 10% homogenate (mechanical ultrasound). After centrifugation at 1000r/minute for 20 minutes under low temperature, 0.4ml supernatant and 3mol/L K2CO3 solution were mixed to adjust the pH to neutral. After centrifugation at 3000r/minute for 5 minutes (under 4° C), the supernatant was dealt with 0.22um filter membrane; then, 10uL supernatant were injected. According to the standard curve equation, the ATP content in the hippocampus was calculated.

### Statistical analysis

An independent samples t-test (data was normal distribution), nonparametric Mann-Whitney U test (data was abnormal distribution), or one-way analysis of variance (one-way ANOVA) was performed when appropriate. If a significant difference was found in one-way ANOVA, Tamhane’s T2 or Bonferroni post-hoc test was used to find out which two groups significantly differed according to the equal variance criterion. The variances between the groups were calculated using Levene’s test. SPSS 19.0 was used to do all analyses, and all tests were two-sided. A p-value<0.05 was considered statistically significant.
